# LI-RADS category 5 hepatocellular carcinoma: preoperative gadoxetic acid–enhanced MRI for early recurrence risk stratification after curative resection

**DOI:** 10.1007/s00330-020-07303-9

**Published:** 2020-10-01

**Authors:** Hong Wei, Hanyu Jiang, Tianying Zheng, Zhen Zhang, Caiwei Yang, Zheng Ye, Ting Duan, Bin Song

**Affiliations:** Department of Radiology, West China Hospital of Sichuan University, No. 37 Guoxue Xiang, Chengdu, 610041 Sichuan China

**Keywords:** Carcinoma, hepatocellular, Liver, Prognosis, Magnetic resonance imaging, Gadoxetic acid

## Abstract

**Objectives:**

To explore the role of preoperative gadoxetic acid–enhanced MRI in stratifying the risk of early recurrence in patients with LR-5 hepatocellular carcinoma (HCC) by LI-RADS v2018 after curative resection.

**Methods:**

Between July 2015 and August 2018, this study evaluated consecutive treatment-naïve at-risk LR-5 HCC patients who underwent gadoxetic acid–enhanced MRI examination within 2 weeks before curative resection. The Cox regression analysis was performed to identify potential predictors of early recurrence. Disease-free survival (DFS) rates were analyzed and compared by using the Kaplan-Meier method and log-rank tests.

**Results:**

Fifty-three of 103 (51.5%) patients experienced early recurrence. Three MRI findings were significantly associated with early recurrence: corona enhancement (hazard ratio [HR]: 2.116; *p* = 0.013), peritumoral hypointensity on hepatobiliary phase (HBP) (HR: 2.262; *p* = 0.007), and satellite nodule (HR: 2.777; *p* = 0.005). An additional risk factor was AFP level > 400 ng/mL (HR: 1.975; *p* = 0.016). Based on the number of MRI predictors, LR-5 HCC patients were stratified into three subgroups: LR-5a (60/103; no predictor), LR-5b (26/103; one predictor), and LR-5c (17/103; two or three predictors), with low, medium, and high risk of early recurrence, respectively. The 2-year DFS rate of LR-5a, LR-5b, and LR-5c patients was 65.0%, 38.5%, and 5.9%, respectively, while the corresponding median DFS was undefined, 17.1 months, and 5.1 months, respectively (*p* < 0.001).

**Conclusions:**

In at-risk LR-5 HCC patients, corona enhancement, peritumoral hypointensity on HBP, and satellite nodule could be used to preoperatively stratify the risk of early recurrence after hepatectomy.

**Key Points:**

• *Corona enhancement, peritumoral hypointensity on HBP, satellite nodule, and serum AFP level > 400 ng/mL were significant predictors of early recurrence in patients with LR-5 HCC after hepatectomy.*

• *Based on the number of predictive MRI findings, LR-5 HCC patients could be preoperatively stratified into three subgroups: LR-5a, LR-5b, and LR-5c, with significantly different risk of early recurrence and disease-free survival.*

• *Preoperative risk stratification is essential for the identification of patients at increased risk of postoperative early recurrence, which may contribute to risk-based personalized management for LR-5 HCC patients.*

**Electronic supplementary material:**

The online version of this article (10.1007/s00330-020-07303-9) contains supplementary material, which is available to authorized users.

## Introduction

Hepatocellular carcinoma (HCC) is the most frequent primary liver malignancy and the third leading cause of cancer-related deaths [1]. Surgical resection is regarded as the first-line treatment option for HCC patients with well-preserved liver function [2]. Nevertheless, ~ 70% of HCC patients develop tumor recurrence within 5 years after surgery [2]. Early recurrence (within 2 years after surgery) is predominantly attributable to the dissemination of the primary HCC and correlated with tumor-related factors (e.g., microvascular invasion, worse differentiation), whereas late recurrence (beyond 2 years after surgery) is more a result of new malignant clones and related to underlying liver conditions (e.g., liver cirrhosis) [2–6]. However, the risk factors of early recurrence, despite critical in terms of treatment, prognosis, and outcome, are mostly evaluated by postoperative pathologic examinations, and their utility in the preoperative context remains limited.

Imaging plays a vital role in the prognostic assessment of HCC patients. Emerging pieces of evidence indicated that preoperative magnetic resonance imaging (MRI) findings, such as rim enhancement, peritumoral hypointensity on hepatobiliary phase (HBP), and nonsmooth tumor margin, were independent risk factors of postoperative recurrence or microvascular invasion (MVI)—a potent risk factor of early recurrence of HCC [7–12]. Although promising, independent validations of these imaging features remain warranted.

Initially released in 2011, the Liver Imaging Reporting and Data System (LI-RADS) was developed to standardize the imaging diagnosis of HCC in at-risk patients [13]. According to the probability of HCC, LI-RADS assigns liver observations in high-risk patients with five major categories from definitely benign (LR-1) to definitely HCC (LR-5). Two additional categories, including LR-M and LR-TIV, are used to describe observations probably or definitely malignant but not HCC-specific and definite tumor in vein, respectively [14]. Among these categories, LR-5 can confirm the diagnosis of HCC with nearly 100% specificity [15], which can be treated without the need for pathologic confirmation. However, LR-5 HCCs represent a wide and heterogenous spectrum of tumors with distinct clinical outcomes, and not all LR-5 HCC patients with resectable tumors, those at increased risk of early recurrence, in particular, would benefit from curative resection. Considering that LI-RADS has been widely used in routine clinical practice, accurate preoperative risk stratification of early recurrence for LR-5 HCC patients is essential for clinical decision-making and patient management. To our knowledge, however, the utility of standardized imaging features defined by LI-RADS in predicting postoperative early recurrence and stratifying disease-free survival (DFS) of patients with LR-5 HCC has not been studied.

Therefore, the purpose of this study was to explore the role of preoperative gadoxetic acid–enhanced MRI features, particularly LI-RADS v2018 imaging features, in predicting early recurrence and stratifying DFS of patients with LR-5 HCC after curative resection.

## Materials and methods

### Patients

**Fig. 1:**
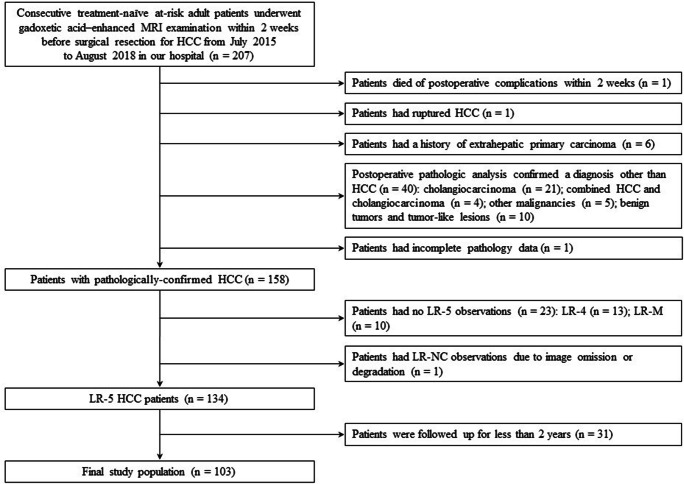
Flow chart of the study population. HCC, hepatocellular carcinoma; NC, not categorizable

This single-center study used data from a prospectively collected observational cohort (Clinical trial registration no: ChiCTR1900026668) and was approved by our institutional review board. Written informed consent was waived. Between July 2015 and August 2018, 207 consecutive treatment-naïve patients with Child-Pugh class A at high-risk for developing HCC (i.e., those with cirrhosis or chronic hepatitis B virus infection) who underwent 3.0-T gadoxetic acid–enhanced MRI within 2 weeks before curative resection were included. Patients were excluded from the study if they (a) died of postoperative complications within 2 weeks (*n* = 1), (b) had ruptured HCCs (*n* = 1), (c) had a history of extrahepatic primary carcinoma (*n* = 6), (d) had non-HCC hepatic tumors confirmed by postoperative pathology (*n* = 40), (e) had incomplete pathology data (*n* = 1), (f) had no LR-5 observations (*n* = 23) or had LR-NC observations (*n* = 1) due to image omission or degradation in accordance with LI-RADS v2018 [14], and (g) were followed up for less than 2 years (*n* = 31) after surgery. Thus, our final study population consisted of 103 patients with pathologically confirmed LR-5 HCCs (Fig. 1).

Clinical information, laboratory data, and pathologic reports were retrieved from electronic medical records. All patients underwent curative resection (R0, defined as complete macroscopic removal of the tumor with a negative margin confirmed by histological examination) after adequate assessment of liver functional reserve, tumor extension, and patient will. The median time between MRI examination and surgery was 2 days (range, 0–12 days).

### MRI examination

All MRI examinations were performed by using a 3.0-T MRI system (Magnetom Skyra, Siemens Healthineers) equipped with an 18-channel body array coil. MRI sequences and parameters are listed in Supplementary Material 1. In- and opposed-phase images were initially obtained by using a T1-weighted two-dimensional gradient-recalled-echo sequence. Dynamic images were obtained before and after contrast agent administration via a T1-weighted three-dimensional gradient-echo sequence in the arterial phase (bolus triggering, 7 s after the peak enhancement of celiac trunk), portal venous phase (60–70 s after contrast agent injection), and transitional phase (180 s after contrast agent injection). For dynamic imaging, 0.1 mL/kg (0.025 mmol/kg) of gadoxetic acid disodium (Primovist; Bayer Schering Pharma) was injected, followed by a 30 mL of 0.9% saline flush at a rate of 2.0 mL/s. Hepatobiliary phase (HBP) images were obtained 20 min after the injection of the contrast agent with the same sequence as that used for the pre- and postcontrast dynamic images. During the interval between transitional and HBP imaging, breath-hold fat-suppressed fast spin-echo T2-weighted imaging was obtained. Diffusion-weighted images were also obtained by using a navigator-triggered technique at *b* values of 0, 50, 500, 800, 1000, and 1200 s/mm^2^ and the corresponding apparent diffusion coefficient maps based on *b* values 0 and 1200 s/mm^2^ were reconstructed.

### Image analysis

Preoperative MR images were retrieved from the Picture Archiving and Communication System and reviewed by two independent radiologists (J. H. Y. and Q.Y., both with 7 years of experience in abdominal MRI) who were blinded to the clinicopathologic and follow-up information. To resolve discrepancies between the two reviewers, all MR images were reassessed together with a third, more experienced radiologist (L.X.J., with 11 years of experience in abdominal MRI) until a consensus was reached.

For each patient at-risk for HCC, the imaging features and LI-RADS categories according to LI-RADS v2018 [14], along with the tumor size, number, and presence or absence of tumor in vein, were evaluated. The following non-LI-RADS imaging features previously reported to be predictive of recurrence or MVI of HCC were also evaluated: non-smooth tumor margin [12], peritumoral hypointensity on HBP [11], incomplete tumor capsule [16], and satellite nodule [17]. The definition of each assessed MR imaging feature is summarized in Supplementary Material 2.

HCC was considered single when nodules close to the primary tumor were designated as satellite nodules; otherwise, HCCs were considered multiple. For patients with multiple tumors, all measurable observations were assessed and the largest observation was selected as the representative for statistical analysis.

### Follow-up surveillance after surgical resection

Postoperative follow-up was performed with contrast-enhanced computed tomography (CT), MRI, or ultrasound, initially at 1 month after surgery and every 3 months thereafter. In addition, monthly blood tests for serum alpha-fetoprotein (AFP) level were obtained.

Early recurrence was defined as intrahepatic and/or extrahepatic recurrence within 2 years after resection of HCC. Recurrence was suspected when the serum AFP levels were highly elevated or new hepatic lesions were observed on ultrasound. Recurrence was established by radiologic evidence (CT or MRI) of new tumors. Increasing serum AFP levels alone without the evidence of new malignancies did not indicate recurrence. All patients were followed until death, early recurrence, or for at least 2 years after curative resection. DFS was defined as the interval from the date of surgery to that of tumor recurrence within 2 years (event), or to that of the last follow-up without early recurrence (censored).

### Statistical analysis

Categorical variables, reported as absolute numbers and rates in percentages, were compared by using the chi-square test, Fisher exact test, or Kruskal–Wallis *H* test, where applicable, whereas continuous variables, expressed as mean ± standard deviation or median (range), were compared by using two-sample *t* test or Mann–Whitney *U* test, where applicable.

DFS was assessed and compared by using the Kaplan–Meier method and log-rank tests. A univariate Cox proportional hazards model was used to assess significant MR imaging findings and clinical factors associated with early recurrence. All variables with *p* < 0.1 at univariate analysis were included for multivariate analysis using a stepwise Cox hazards regression model (forward LR). All statistical analyses were conducted by using SPSS software (version 22.0, IBM). A *p* < 0.05 was considered statistically significant.

## Results

### Baseline patient characteristics

Key clinicopathologic and MR imaging characteristics of the study population are summarized in Table 1. There were no significant differences between early recurrence present (*n* = 53) and absent (*n* = 50) groups in terms of the most assessed characteristics. For clinical parameters, patients with early recurrence showed higher serum AFP levels (*p* = 0.026) and more advanced BCLC stages than those without early recurrence (*p* = 0.004).

**Table 1:** Clinicopathologic and MR imaging characteristics of the study population

Characteristic	Total* (*n* = 103)	Early recurrence absent* (*n* = 50)	Early recurrence present* (*n* = 53)	*p* value
Age (years)^†^	51.0 ± 12.0	53.1 ± 11.3	49.0 ± 12.4	0.078
Sex				0.559
Female	21 (20.4)	9 (18.0)	12 (22.6)	
Male	82 (79.6)	41 (82.0)	41 (77.4)	
HBsAg				0.353
Negative	17 (16.5)	10 (20.0)	7 (13.2)	
Positive	86 (83.5)	40 (80.0)	46 (86.8)	
Serum ALT level (IU/L)^†^	37.0 (11.0–620.0)	35.0 (11.0–620.0)	40.0 (11.0–225.0)	0.533
Serum AST level (IU/L)^†^	39.0 (18.0–243.0)	36.5 (19.0–176.0)	42.0 (18.0–243.0)	0.438
Serum TBIL level (μmol/L)^†^	13.9 (4.2–54.2)	13.7 (7.7–54.2)	14.6 (4.2–31.1)	0.642
Prothrombin time (s)^†^	12.3 ± 1.0	12.2 ± 1.1	12.3 ± 1.0	0.476
Platelet count (× 10^9/L)^†^	148.0 (40.0–492.0)	147.5 (58.0–492.0)	152.0 (40.0–367.0)	0.518
Serum AFP level				0.026
≤ 400 ng/mL	65 (63.1)	37 (74.0)	28 (52.8)	
> 400 ng/mL	38 (36.9)	13 (26.0)	25 (47.2)	
BCLC stage				0.004
0–A	35 (34.0)	23 (46.0)	12 (22.6)	
AB^††^	38 (36.9)	18 (36.0)	20 (37.7)	
B	8 (7.8)	3 (6.0)	5 (9.4)	
C	22 (21.4)	6 (12.0)	16 (30.2)	
Pathologic features				
Tumor differentiation				0.022
Well or moderately differentiated	54 (52.4)	32 (64.0)	22 (41.5)	
Poorly differentiated	49 (47.6)	18 (36.0)	31 (58.5)	
Microvascular invasion				0.042
Absent	75 (72.8)	41 (82.0)	34 (64.2)	
Present	28 (27.2)	9 (18.0)	19 (35.8)	
Serosal invasion				0.036
Absent	55 (53.4)	32 (64.0)	23 (43.4)	
Present	48 (46.6)	18 (36.0)	30 (56.6)	
Satellite nodule				0.045
Absent	94 (91.3)	49 (98.0)	45 (84.9)	
Present	9 (8.7)	1 (2.0)	8 (15.1)	
Cirrhosis				0.098
Absent	64 (62.1)	27 (54.0)	37 (69.8)	
Present	39 (37.9)	23 (46.0)	16 (30.2)	
MR imaging features				
Tumor size (cm)^†^	6.3 ± 3.0	5.4 ± 2.5	7.2 ± 3.3	0.002
No. of tumor				0.049
Single	90 (87.4)	47 (94.0)	43 (81.1)	
Multiple	13 (12.6)	3 (6.0)	10 (18.9)	
LI-RADS major features				
Tumor size ≥ 20 mm				0.325
Absent	5 (4.9)	4 (8.0)	1 (1.9)	
Present	98 (95.1)	46 (92.0)	52 (98.1)	
Non-rim arterial phase hyperenhancement				…
Absent	0 (0.0)	0 (0.0)	0 (0.0)	
Present	103 (100.0)	50 (100.0)	53 (100.0)	
Non-peripheral washout				…
Absent	0 (0.0)	0 (0.0)	0 (0.0)	
Present	103 (100.0)	50 (100.0)	53 (100.0)	
Enhancing capsule				0.205
Absent	31 (30.1)	18 (36.0)	13 (24.5)	
Present	72 (69.9)	32 (64.0)	40 (75.5)	
LI-RADS ancillary features (favoring HCC in particular)				
Non-enhancing capsule				0.055
Absent	84 (81.6)	37 (74.0)	47 (88.7)	
Present	19 (18.4)	13 (26.0)	6 (11.3)	
Nodule-in-nodule architecture				0.099
Absent	45 (43.7)	26 (52.0)	19 (35.8)	
Present	58 (56.3)	24 (48.0)	34 (64.2)	
Mosaic architecture				0.280
Absent	26 (25.2)	15 (30.0)	11 (20.8)	
Present	77 (74.8)	35 (70.0)	42 (79.2)	
Fat in mass, more than adjacent liver				0.110
Absent	81 (78.6)	36 (72.0)	45 (84.9)	
Present	22 (21.4)	14 (28.0)	8 (15.1)	
Blood products in mass				0.197
Absent	53 (51.5)	29 (58.0)	24 (45.3)	
Present	50 (48.5)	21 (42.0)	29 (54.7)	
LI-RADS ancillary features (favoring malignancy, not HCC in particular)				
Transitional phase hypointensity				1.000
Absent	2 (1.9)	1 (2.0)	1 (1.9)	
Present	101 (98.1)	49 (98.0)	52 (98.1)	
Restricted diffusion				…
Absent	0 (0.0)	0 (0.0)	0 (0.0)	
Present	103 (100.0)	50 (100.0)	53 (100.0)	
Mild-moderate T2 hyperintensity				…
Absent	0 (0.0)	0 (0.0)	0 (0.0)	
Present	103 (100.0)	50 (100.0)	53 (100.0)	
Corona enhancement				0.001
Absent	75 (72.8)	44 (88.0)	31 (58.5)	
Present	28 (27.2)	6 (12.0)	22 (41.5)	
Fat sparing in solid mass				0.262
Absent	100 (97.1)	50 (100.0)	50 (94.3)	
Present	3 (2.9)	0 (0.0)	3 (5.7)	
Hepatobiliary phase hypointensity				0.569
Absent	4 (3.9)	3 (6.0)	1 (1.9)	
Present	99 (96.1)	47 (94.0)	52 (98.1)	
Iron sparing in solid mass				0.485
Absent	102 (99.0)	49 (98.0)	53 (100.0)	
Present	1 (1.0)	1 (2.0)	0 (0.0)	
Tumor in vein				0.024
Absent	81 (78.6)	44 (88.0)	37 (69.8)	
Present	22 (21.4)	6 (12.0)	16 (30.2)	
Non-LIRADS imaging features				
Non-smooth tumor margin				0.286
Absent	48 (46.6)	26 (52.0)	22 (41.5)	
Present	55 (53.4)	24 (48.0)	31 (58.5)	
Peritumoral hypointensity on HBP				0.002
Absent	79 (76.7)	45 (90.0)	34 (64.2)	
Present	24 (23.3)	5 (10.0)	19 (35.8)	
Incomplete tumor capsule				0.205
Absent	49 (47.6)	27 (54.0)	22 (41.5)	
Present	54 (52.4)	23 (46.0)	31 (58.5)	
Satellite nodule				0.003
Absent	91 (88.3)	49 (98.0)	42 (79.2)	
Present Single	12 (11.7) 7 (58.3)	1 (2.0) 0 (0.0)	11 (20.8) 7 (63.6)	0.417 0.865
Multiple	5 (41.7)	1 (100.0)	4 (36.4)	
Size (cm)^†^	1.4 ± 0.4	1.3	1.4 ± 0.4	

*AFP* alpha-fetoprotein, *ALT* alanine aminotransferase, *AST* aspartate aminotransferase, *BCLC* Barcelona Clinic Liver Cancer, *HBP* hepatobiliary phase, *HCC* hepatocellular carcinoma, *LI-RADS* Liver Imaging Reporting and Data System, *TBIL* total bilirubin

*Unless otherwise indicated, data are number of patients, with percentage in parentheses. Categorical variables were compared by using the chi-square test, Fisher exact test, or Kruskal–Wallis *H* test

^†^Data are continuous variables, reported as mean ± standard deviation or median (range), and were compared by using the two-sample *t* test or Mann–Whitney *U* test

^††^BCLC stage AB was defined as a single large (> 5 cm) HCC with Child-Pugh classification A–B and performance status 0 and without vascular invasion

With regard to pathologic features, patients with early recurrence had more poorly differentiated tumors (58.5% vs. 36.0%, *p* = 0.022), higher proportion of MVI (35.8% vs. 18.0%, *p* = 0.042), serosal invasion (56.6% vs. 36.0%, *p* = 0.036), and satellite nodule (15.1% vs. 2.0%, *p* = 0.045) than those without early recurrence.

Among MR imaging features, mean tumor size of patients with early recurrence was larger than that of patients without early recurrence (7.2 cm vs. 5.4 cm, *p* = 0.002). Multiple tumors (18.9% vs. 6.0%, *p* = 0.049), corona enhancement (41.5% vs. 12.0%, *p* = 0.001), tumor in vein (30.2% vs. 12.0%, *p* = 0.024), peritumoral hypointensity on HBP (35.8% vs. 10.0%, *p* = 0.002), and satellite nodule (20.8% vs. 2.0%, *p* = 0.003) were significantly more frequent in patients with early recurrence compared with those without early recurrence.

### Early recurrence after curative resection

During a median follow-up period of 22.1 months (range, 1.0–46.5 months), tumor early recurrence was observed in 51.5% (53/103) patients. Among patients with early recurrence, 83.0% (44/53) experienced intrahepatic recurrence, 7.5% (4/53) experienced extrahepatic recurrence, and the remaining 9.4% (5/53) experienced combined intra- and extrahepatic recurrence. The distant metastasis sites included lungs (*n* = 5), bone (*n* = 2), lymph nodes (*n* = 1), and adrenal glands (*n* = 1).

### Prediction of early recurrence

Of 196 measurable HCC nodules, 158 (80.6%) were categorized as LR-5. The two reviewers agreed on the LR-5 category of most cases (150 of 158 nodules, 94.9%) and reached a consensus through reassessment and discussion of the remaining 8 nodules with the third reviewer.

Tumor size, multiple tumors, non-enhancing capsule, nodule-in-nodule architecture, corona enhancement, fat sparing in solid mass, tumor in vein, peritumoral hypointensity on HBP, satellite nodule, serum AFP level > 400 ng/mL, and BCLC stage were identified as significant predictors of early recurrence at univariate analysis. However, only corona enhancement (hazard ratio [HR]: 2.116; *p* = 0.013), peritumoral hypointensity on HBP (HR: 2.262; *p* = 0.007), satellite nodule (HR: 2.777; *p* = 0.005), and serum AFP level > 400 ng/mL (HR: 1.975; *p* = 0.016) were significant predictors of early recurrence at multivariate analysis (Table 2).

**Table 2:** Univariate and multivariate analyses for early recurrence

	Univariate analysis			Multivariate analysis		
Parameter	HR	95% CI	*p* value	HR	95% CI	*p* value
Tumor size	1.188	1.085, 1.301	< 0.001	…	…	…
Multiple tumors	2.317	1.159, 4.631	0.017	…	…	…
LI-RADS major features						
Tumor size ≥ 20 mm	3.227	0.446, 23.349	0.246	…	…	…
Non-rim arterial phase hyperenhancement	…	…	…	…	…	…
Non-peripheral washout	…	…	…	…	…	…
Enhancing capsule	1.382	0.739, 2.584	0.311	…	…	…
LI-RADS ancillary features (favoring HCC in particular)						
Non-enhancing capsule	0.475	0.203, 1.112	0.086	…	…	…
Nodule-in-nodule architecture	1.637	0.933, 2.873	0.086	…	…	…
Mosaic architecture	1.461	0.752, 2.838	0.264	…	…	…
Fat in mass, more than adjacent liver	0.562	0.265, 1.194	0.134	…	…	…
Blood products in mass	1.496	0.871, 2.571	0.145	…	…	…
LI-RADS ancillary features (favoring malignancy, not HCC in particular)						
Transitional phase hypointensity	1.162	0.161, 8.413	0.882	…	…	…
Restricted diffusion	…	…	…	…	…	…
Mild-moderate T2 hyperintensity	…	…	…	…	…	…
Corona enhancement	2.880	1.656, 5.010	< 0.001	2.116	1.172, 3.820	0.013
Fat sparing in solid mass	2.738	0.843, 8.892	0.094	…	…	…
Hepatobiliary phase hypointensity	2.430	0.336, 17.583	0.379	…	…	…
Iron sparing in solid mass	0.049	0.000, 1631.454	0.570	…	…	…
Tumor in vein	2.332	1.290, 4.217	0.005	…	…	…
Non-LIRADS imaging features						
Non-smooth tumor margin	1.462	0.846, 2.526	0.174	…	…	…
Peritumoral hypointensity on HBP	2.759	1.566, 4.859	< 0.001	2.262	1.248, 4.100	0.007
Incomplete tumor capsule	1.442	0.834, 2.492	0.190	…	…	…
Satellite nodule	3.490	1.764, 6.905	< 0.001	2.777	1.369, 5.634	0.005
Clinical factors						
Serum AFP level > 400 ng/mL	2.107	1.224, 3.628	0.007	1.975	1.136, 3.437	0.016
BCLC stage						
0–A^†^	…	…	…	…	…	…
AB^††^	1.762	0.861, 3.605	0.121	…	…	…
B	2.428	0.855, 6.900	0.096	…	…	…
C	3.407	1.601, 7.248	0.001	…	…	…

Variables with *p* < 0.1 in univariate analysis were applied to multivariate analysis using a stepwise Cox proportional hazards regression model

*AFP* alpha-fetoprotein, *BCLC* Barcelona Clinic Liver Cancer, *CI* confidence interval, *HBP* hepatobiliary phase, *HCC* hepatocellular carcinoma, *HR* hazard ratio, *LI-RADS* Liver Imaging Reporting and Data System

^†^Used as the reference category

^††^BCLC stage AB was defined as a single large (> 5 cm) HCC with Child-Pugh class A–B and performance status 0 and without vascular invasion

The sensitivity, specificity, PPV, NPV, and accuracy of each indicator predictive of early recurrence are summarized in Table 3. In addition, preoperative gadoxetic acid–enhanced MR imaging features for predicting early recurrence of HCC after surgical resection in published studies [7–9, 12, 18–22] are shown in Table 4.

**Table 3:** Diagnostic performance of each significant factor in prediction of early recurrence

Characteristic	Sensitivity (%)	Specificity (%)	PPV (%)	NPV (%)	Accuracy (%)
MR imaging finding					
Corona enhancement	41.5 (22/53)	88.0 (44/50)	78.6 (22/28)	58.7 (44/75)	64.1 (66/103)
Peritumoral hypointensity on HBP	35.8 (19/53)	90.0 (45/50)	79.2 (19/24)	57.0 (45/79)	62.1 (64/103)
Satellite nodule	20.8 (11/53)	98.0 (49/50)	91.7 (11/12)	53.8 (49/91)	58.3 (60/103)
Tumor marker					
Serum AFP level > 400 ng/mL	47.2 (25/53)	74.0 (37/50)	65.8 (25/38)	56.9 (37/65)	60.2 (62/103)

*AFP* alpha-fetoprotein, *HBP* hepatobiliary phase, *MR* magnetic resonance, *NPV* negative predictive value, *PPV* positive predictive value

**Table 4:** Preoperative gadoxetic acid–enhanced MR imaging findings for predicting early recurrence after surgical resection of HCC in published studies

Author	Year	No. of patients	Predictive MR imaging findings for early recurrence^*^
Ahn SJ et al [7]	2019	179	Satellite nodule, peritumoral hypointensity on HBP, absence of capsule, and texture parameter
Lee S et al [8]	2017	197	Arterial peritumoral enhancement, non-smooth tumor margin, and peritumoral hypointensity on HBP
An C et al [9]	2015	268	Rim enhancement, peritumoral parenchymal enhancement, satellite nodule, and tumor size
Arrizumi S et al [12]^†^	2011	61	Non-smooth tumor margin on HBP
Cha DI et al [18]	2020	549	Arterial rim enhancement of the tumor, non-hypervascular hepatobiliary hypointense nodules, and tumor size
Zhang L et al [19]^†^	2019	82	Corona enhancement and irregular tumor margin
Zhang Z et al [20]^†^	2019	155	Radiomics score, gross vascular invasion, and non-smooth tumor margin
Kim S et al [21]	2019	167	Radiomics features with 3-mm border extension
Hectors SJ et al [22]^†^	2020	48	Texture features and tumor size
Wei H et al (this study)	2020	103	Corona enhancement, peritumoral hypointensity on HBP, and satellite nodule

*HBP* hepatobiliary phase, *HCC* hepatocellular carcinoma, *MR* magnetic resonance

*Unless otherwise indicated, early recurrence was defined as recurrence within 2 years after resection of HCC

^†^Early recurrence was defined as recurrence within 1 year after resection of HCC

### Imaging features for DFS stratification

**Fig. 2:**
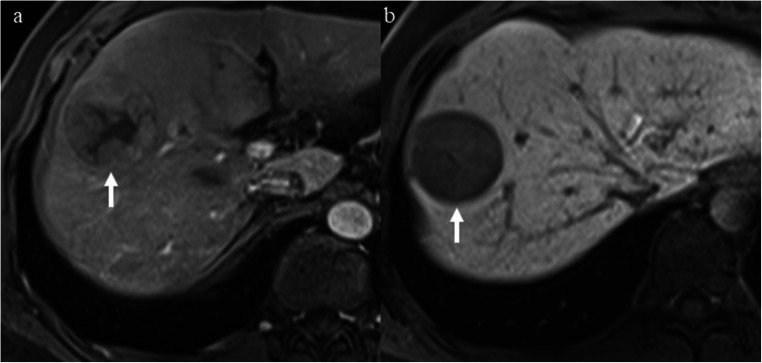
A 47-year-old man with a 4.5-cm moderately differentiated LR-5 HCC in hepatic segment VIII and the serum AFP level was higher than 1210 ng/mL. **a** Mass shows non-rim hyperenhancement and mosaic architecture (arrow) on late arterial phase. **b** Mass shows hypointensity and non-smooth tumor margin (arrow) on hepatobiliary phase. This patient had none of the predictive MR imaging findings for early recurrence and was categorized as LR-5a. There was no early recurrence during follow-up after curative resection. The disease-free survival was 37.6 months

**Fig. 3:**
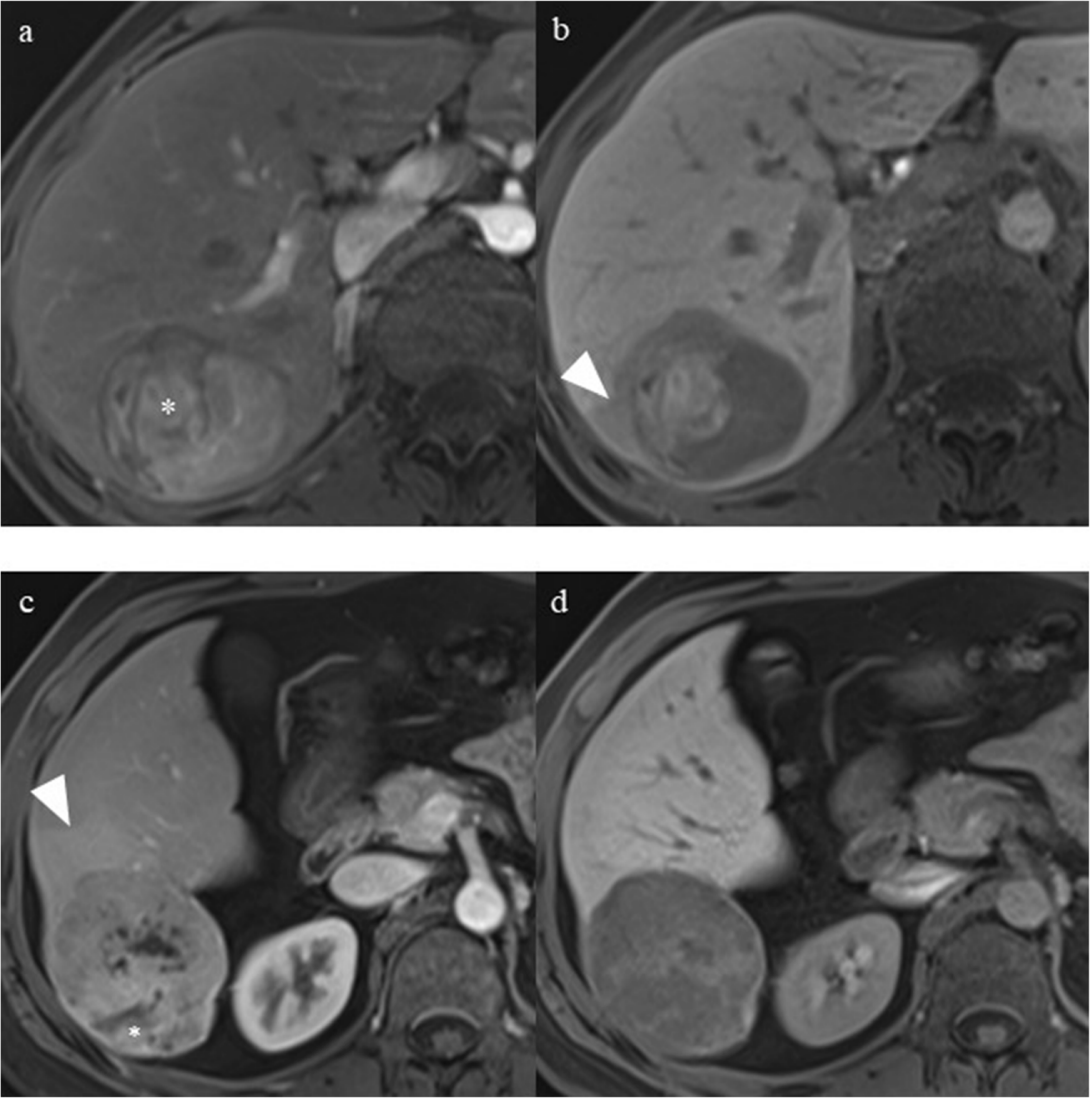
**a**, **b** A 47-year-old woman with a 5.2-cm poorly differentiated LR-5 HCC in hepatic segment VI and serum AFP level of 1.38 ng/mL. **a** Mass shows non-rim hyperenhancement, nodule-in-nodule (asterisk), and mosaic architecture on late arterial phase. **b** Mass shows mixed intensity and peritumoral hypointensity (arrowhead) on hepatobiliary phase (HBP). This patient had one of the predictive MR imaging findings (peritumoral hypointensity on HBP) for early recurrence and was categorized as LR-5b. Early recurrence occurred in the liver during follow-up after curative resection. The disease-free survival was 23.7 months. **c**, **d** A 29-year-old man with a 7.0-cm moderately differentiated LR-5 HCC in hepatic segment VI and the serum AFP level was higher than 1210 ng/mL. **c** Mass shows non-rim hyperenhancement, nodule-in-nodule (asterisk), mosaic architecture, and corona enhancement (arrowhead) on late arterial phase. **d** Mass shows hypointensity and non-smooth tumor margin on hepatobiliary phase. This patient had one of the predictive MR imaging findings (corona enhancement) for early recurrence and was categorized as LR-5b. Early recurrence occurred in the liver during follow-up after curative resection. The disease-free survival was 8.5 months

**Fig. 4:**
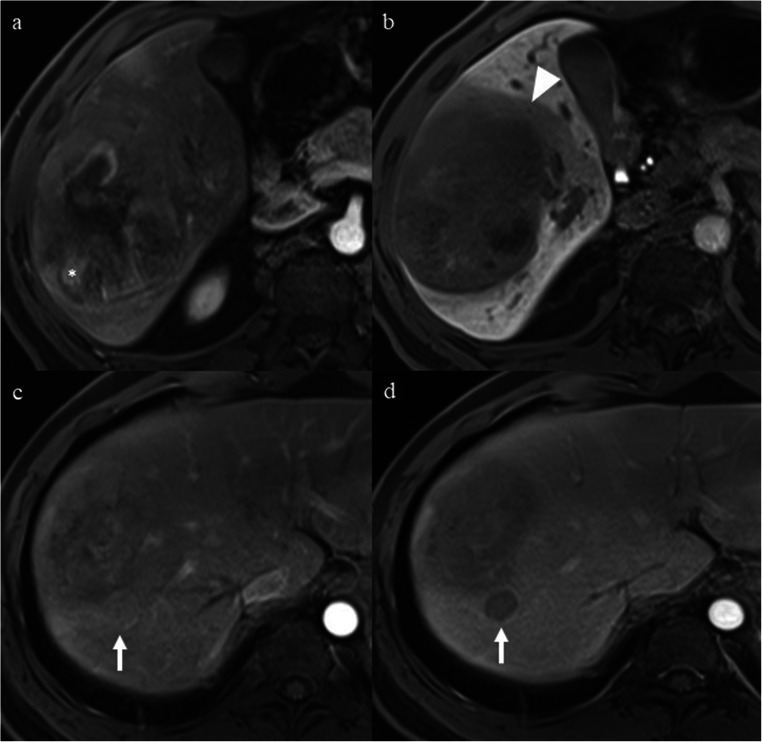
A 38-year-old man with a 10.0-cm poorly differentiated LR-5 HCC in hepatic segment V-VIII and serum AFP level of 5.06 ng/mL. **a** Mass shows non-rim hyperenhancement, nodule-in-nodule (asterisk), and mosaic architecture on late arterial phase. **b** Mass shows hypointensity, non-smooth tumor margin, and peritumoral hypointensity (arrowhead) on hepatobiliary phase. **c** A 1.7-cm satellite nodule (arrow) located in the peritumoral parenchyma shows non-rim hyperenhancement on late arterial phase. **d** The satellite nodule (arrow) shows non-peripheral “washout” on portal venous phase. This patient had two of the predictive MR imaging findings (peritumoral hypointensity on HBP and satellite nodule) for early recurrence and was categorized as LR-5c. Early recurrence occurred in the liver during follow-up after curative resection. The disease-free survival was 3.7 months

**Fig. 5:**
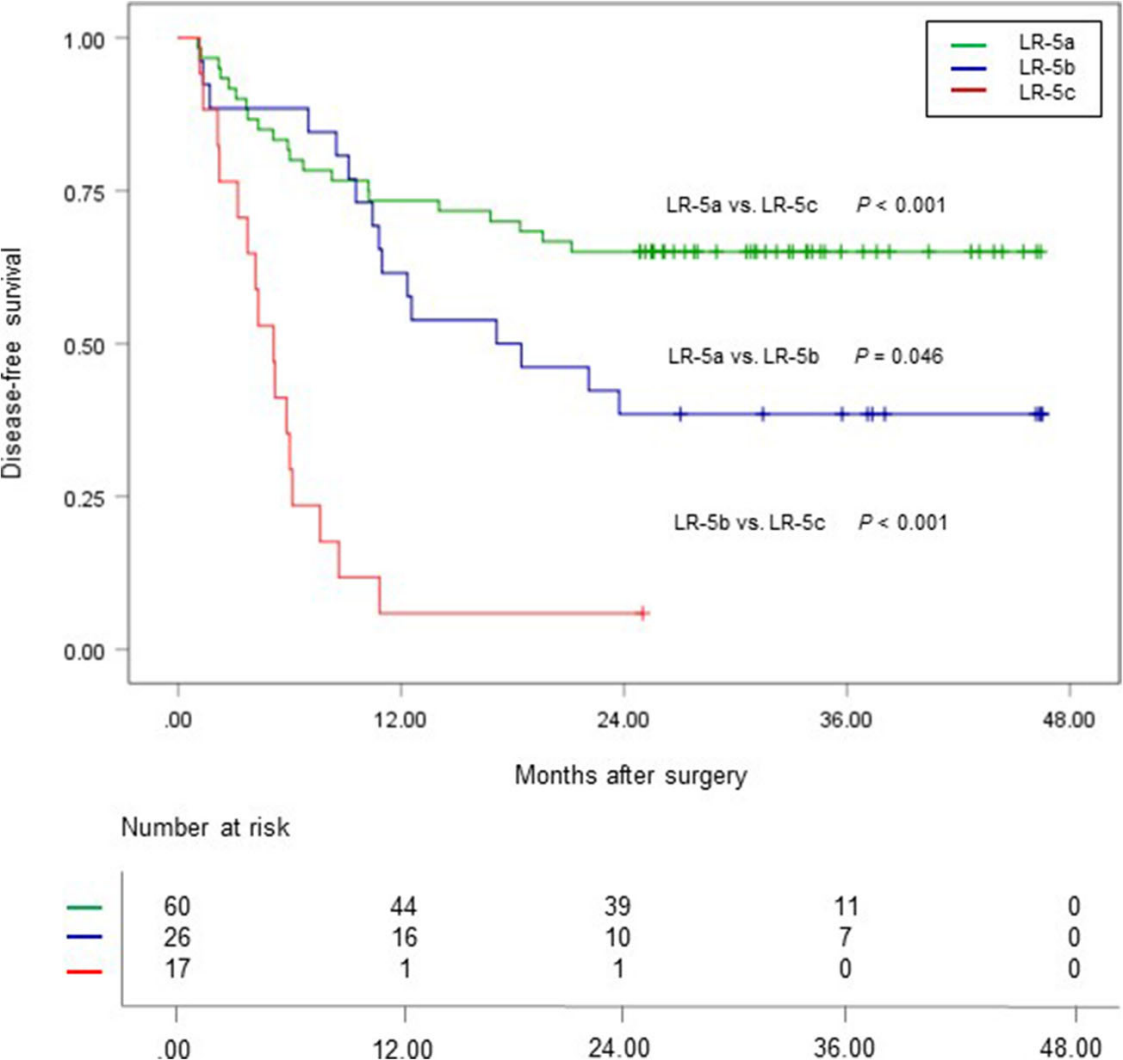
Disease-free survival (DFS) curves of LR-5a (green), LR-5b (blue), and LR-5c (red) subgroups, with LR-5 HCC patients having no, one, and two or three significant MR imaging findings for predicting early recurrence, respectively. DFS differed significantly among three subgroups of LR-5 HCC patients according to the Kaplan–Meier method and log-rank tests

Based on the number of MR imaging predictors, 103 LR-5 HCC patients were stratified into three subgroups according to their risk of early recurrence: LR-5a (low-risk patients, 60/103), LR-5b (medium-risk patients, 26/103), and LR-5c (high-risk patients, 17/103) with no, one, or no less than two imaging predictors of early recurrence, respectively (Figs. 2, 3, and 4). The 2-year DFS rate of LR-5a, LR-5b, and LR-5c HCC patients was 65.0% (95% confidence interval [CI]: 54.0%, 78.3%), 38.5% (95% CI: 23.7%, 62.5%), and 5.9% (95% CI: 0.9%, 39.4%), respectively (*p* < 0.001); while the corresponding median DFS was undefined, 17.1 months (95% CI: 4.9 months, 29.3 months) and 5.1 months (95% CI: 3.7 months, 6.5 months), respectively (*p* < 0.001) (Fig. 5).

**Fig. 6:**
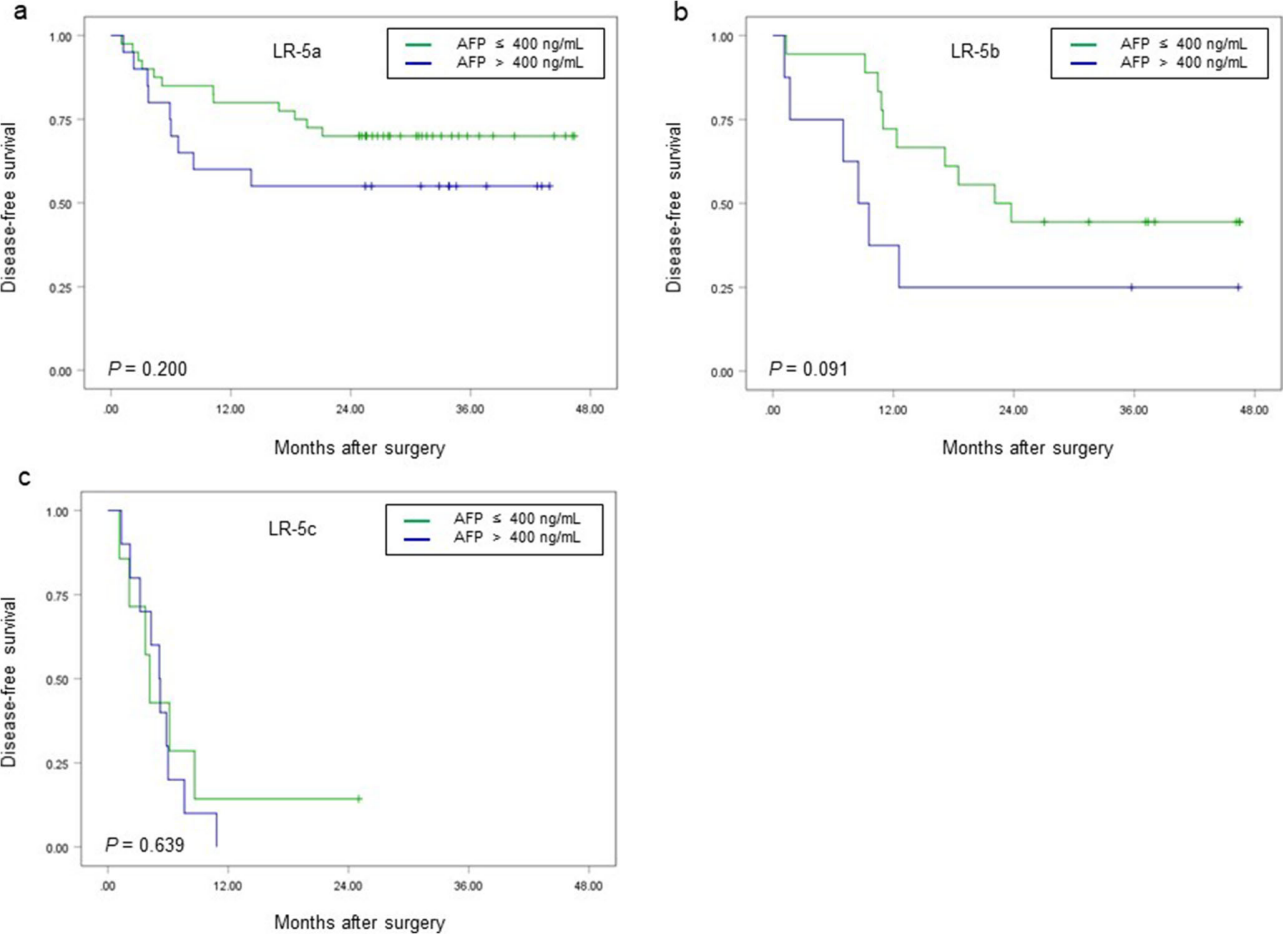
Comparison of disease-free survival (DFS) according to the serum AFP level in LR-5a (**a**), LR-5b (**b**), and LR-5c (**c**) subgroups. There were no significant differences in DFS between patients with serum AFP level > 400 ng/mL and those with serum AFP level ≤ 400 ng/mL for three subgroups

Furthermore, for LR-5a, LR-5b, and LR-5c subgroups, patients with serum AFP level > 400 ng/mL had lower 2-year DFS rate than those with serum AFP level ≤ 400 ng/mL, but the differences were not significant (LR-5a: 55.0% (95% CI: 37.0%, 81.8%) vs. 70.0% (95% CI: 57.1%, 85.7%), *p* = 0.200; LR-5b: 25.0% (95% CI: 7.5%, 83.0%) vs. 44.4% (95% CI: 26.5%, 74.5%), *p* = 0.091; LR-5c: 0.0% vs. 14.3% (95% CI: 2.3%, 87.7%), *p* = 0.639) (Fig. 6).

### Pathologic features in relation to predictive MR imaging findings

Pathologic features in relation to predictive MR imaging findings are shown in Table 5. HCCs with corona enhancement showed worse tumor differentiation than those without this finding (67.9% vs. 40.0% poorly differentiated tumors, *p* = 0.012). HCCs with peritumoral hypointensity on HBP showed a significantly higher proportion of MVI compared with those without this finding (50.0% vs. 20.3%, *p* = 0.004). HCCs with satellite nodule on MRI showed a significantly higher proportion of pathologically determined satellite nodule than those without this finding (41.7% vs. 4.4%, *p* < 0.001).

**Table 5:** Pathologic features in relation to predictive MR imaging findings

Pathologic feature	Corona enhancement			Peritumoral hypointensity on HBP			Satellite nodule		
	Absent	Present	*p*	Absent	Present	*p*	Absent	Present	*p*
Tumor differentiation			0.012			0.095			0.858
Well or moderately differentiated	45 (60.0)	9 (32.1)		45 (57.0)	9 (37.5)		48 (52.7)	6 (50.0)	
Poorly differentiated	30 (40.0)	19 (67.9)		34 (43.0)	15 (62.5)		43 (47.3)	6 (50.0)	
Microvascular invasion			0.490			0.004			0.870
Absent	56 (74.7)	19 (67.9)		63 (79.7)	12 (50.0)		67 (73.6)	8 (66.7)	
Present	19 (25.3)	9 (32.1)		16 (20.3)	12 (50.0)		24 (26.4)	4 (33.3)	
Serosal invasion			0.190			0.703			0.386
Absent	43 (57.3)	12 (42.9)		43 (54.4)	12 (50.0)		50 (54.9)	5 (41.7)	
Present	32 (42.7)	16 (57.1)		36 (45.6)	12 (50.0)		41 (45.1)	7 (58.3)	
Satellite nodule			0.107			0.247			< 0.001
Absent	71 (94.7)	23 (82.1)		74 (93.7)	20 (83.3)		87 (95.6)	7 (58.3)	
Present	4 (5.3)	5 (17.9)		5 (6.3)	4 (16.7)		4 (4.4)	5 (41.7)	
Cirrhosis			0.465			0.050			0.978
Absent	45 (60.0)	19 (67.9)		45 (57.0)	19 (79.2)		56 (61.5)	8 (66.7)	
Present	30 (40.0)	9 (32.1)		34 (43.0)	5 (20.8)		35 (38.5)	4 (33.3)	

Data are number of patients with percentage in parentheses

*HBP* hepatobiliary phase, *MR* magnetic resonance

## Discussion

The results of this study demonstrated that based on the number of three MR imaging findings (corona enhancement, peritumoral hypointensity on HBP, and satellite nodule), LR-5 HCC patients could be preoperatively stratified into three subgroups: LR-5a, LR-5b, and LR-5c, with significantly different risk of early recurrence. This could be of clinical significance, considering that LR-5 HCC can be treated without the requirement for histologic confirmation. Preoperative risk stratification is essential for clinicians to identify patients at increased risk of postoperative early recurrence, which may contribute to risk-based personalized management, such as considering the use of extended resection or liver transplantation for selected LR-5c HCC patients, or making tailored follow-up plans and subsequent adjuvant treatment strategies for them.

As opposed to most previous studies in which patients were classified into two subgroups (low and high risk) according to their risk of recurrence [7, 9, 10, 18–22], herein, we identified a fraction of medium-risk patients by stratifying LR-5 HCC patients into three subcategories with significantly different early recurrence rate and disease-free survival. Although Qin X et al stratified HCC patients into three subgroups with low, medium, and high recurrence rate by using the parameters of T1 mapping, the recurrence rates were not significantly different between subgroups of high- and medium-risk of recurrence in that study [23]. In addition, our method only requires counting the number of the risk factors, with complicated equations or calculations uninvolved, which enables clinicians to estimate the risk stratification quickly and discuss with patients efficiently before treatment.

Corona enhancement and satellite nodule were significant MR imaging findings for predicting early recurrence of HCC in our study. These results corresponded well with several previous studies [7, 9, 19]. Prior findings suggested that the presence of corona enhancement could indicate the progressed HCC that has a propensity to invade drainage vessels of the tumor, leading to intrahepatic metastases [24–26]. These metastases usually manifested as satellite nodules within the venous drainage area around the tumor, which can lead to vascular invasion and metastases [25, 27]. Likewise, our study also showed that HCCs with corona enhancement had worse tumor differentiation than those without this finding, which is supported by these studies.

Peritumoral hypointensity on HBP was another MR imaging finding predictive of early recurrence of HCC in our study, consistent with previous studies [7, 8]. Peritumoral hypointensity on HBP has been reported as a preoperative biomarker for MVI, a well-validated risk factor of early recurrence of HCC [8, 11]. Similarly, in our study, among three predictive MR imaging findings, MVI is more frequently observed only in HCCs with peritumoral hypointensity on HBP than those without this finding, as with these studies. One possible mechanism underlying the appearance of peritumoral hypointensity on HBP is that peritumoral perfusion changes due to MVI might influence the function of organic anion-transporting polypeptide transporters, leading to a decrease in gadoxetic acid uptake in peritumoral hepatocytes [8]. However, in a small fraction of patients in our study, peritumoral hypointensity on HBP was also accompanied by the presence of hypointense areas in the hepatic segment where the tumors were located. In these cases, we speculated that other potential causes, such as biliary stasis, may have resulted in the appearance of hypointensity in the hepatic segment adjacent to the tumors on HBP. In this context, it might be challenging to determine the exact cause of peritumoral hypointensity on HBP. Currently, few published studies have adequately explored the pathologic causes of peritumoral hypointensity on HBP in HCCs. Further studies looking at this issue are required.

Serum AFP level > 400 ng/mL was an additional significant factor for predicting early recurrence in our study. Zhang Y et al have reported that serum AFP level > 400 ng/mL was related to HCC recurrence after hepatectomy [28], consistent with our result. Nevertheless, in LR-5a, LR-5b, and LR-5c subgroups, the 2-year DFS rates were not significantly different between patients with serum AFP level > 400 ng/mL and those with serum AFP level ≤ 400 ng/mL. This indicates that MR imaging findings are superior to the serum AFP level in predicting early recurrence of HCC.

The LI-RADS major features (except for threshold growth) and ancillary features (except for corona enhancement), and other non-LIRADS imaging features reported to be correlated with postoperative recurrence, such as tumor size [9, 18, 22], multiple tumors [10, 29, 30], non-smooth tumor margin [8, 10, 12, 19, 20], and incomplete tumor capsule [31], did not show significant associations with early recurrence in our study. However, as reported in a recent study, the absence of fat in mass (one LI-RADS ancillary features favoring HCC in particular) was an independent predictor for recurrence in patients with LR-5 HCC [10]. This may be partly due to the different endpoint events between our study and the previous study, in which recurrent HCCs were not distinguished as early and late recurrence. In addition, the different study populations (only LR-5 HCCs were enrolled in our study) and the small proportion of multiple HCCs (12.6%) may be partly responsible for the discrepant results between our study and the prior studies.

Counterintuitively, in our study, tumor in vein (TIV) was not a significant predictor of early recurrence at multivariate analysis. A possible explanation may be that our study population were all selected surgical candidates with resectable tumors. In other words, patients with TIV involving the major blood vessels were excluded. Thus, the less severe TIV of our study population may be inadequate to result in the increased risk of early recurrence.

Intriguingly, BCLC stage was not independently associated with early recurrence in our study. This indicates that the designation of BCLC stage B or C should not be an absolute contraindication for surgery. Surgical resection could be considered for selected patients with BCLC stage B or C after careful assessment of liver functional remnant, tumor expansion, and patient will. However, our findings remain to be validated further since the relationship between BCLC stage and late recurrence was not estimated in our study, and there was a relatively small proportion of BCLC stage B and C patients (29.1%, 30/103) in our study population.

This study had several limitations. First, substantial selection bias could have been introduced due to the retrospective nature, together with the limited sample size. Future studies including prospective multicenter enrollment will be required to validate the results of our study. Second, we only assessed LR-5 observations that were pathologically confirmed as HCC, whereas non-HCC LR-5 observations were not included. However, the pooled percentage of HCC for LR-5 observation has been reported to be 94% [32], suggesting that non-HCC LR-5 observation is infrequent. Finally, in this study, the follow-up time was relatively short and the late recurrence was not assessed. Future studies with longer follow-up period are warranted to evaluate the role of MR imaging features in predicting both early and late HCC recurrence and stratifying long-term survival time.

In conclusion, in at-risk patients with LR-5 HCC, corona enhancement, peritumoral hypointensity on HBP, and satellite nodule can be used to effectively predict early recurrence and preoperatively stratify the DFS after curative resection. This may contribute to the risk-based personalized management for LR-5 HCC patients.

## Electronic supplementary material

ESM 1 (DOCX 31 kb)

## Abbreviations

AFP: Alpha-fetoprotein
CT: Computed tomography
DFS: Disease-free survival
HBP: Hepatobiliary phase
HCC: Hepatocellular carcinoma
LI-RADS/LR: Liver Imaging Reporting and Data System
LR: Likelihood ratio
M: Malignancy
MR: Magentic resonance
MRI: Magnetic resonance imaging
MVI: Microvascular invasion
NC: Not categorizable
NPV: Negative predictive value
PPV: Positive predictive value
TIV: Tumor in vein
