# Supplementary material for: LI-RADS category 5 hepatocellular carcinoma: preoperative gadoxetic acid–enhanced MRI for early recurrence risk stratification after curative resection
Source: Eur Radiol. 2020 Oct 1;31(4):2289–302. doi: 10.1007/s00330-020-07303-9 (PMC7979599; doi:10.1007/s00330-020-07303-9)
Supplement: Supplementary file 1 — (DOCX 31 kb) [file 330_2020_7303_MOESM1_ESM.docx]

Supplementary Material 1 MRI Sequences and Parameters

| Sequence | Fat suppression | TR  (ms) | TE  (ms) | Flip angle | Section thickness (mm) | Matrix size | Field of view (mm^2^) | Acquisition time (s) |
| --- | --- | --- | --- | --- | --- | --- | --- | --- |
| T2-weighted 2D TSE  Coronal T2-weighted HASTE  T1-weighted 3D GRE VIBE  T1-weighted IP and OP imaging  DW single-shot spin-echo EPI | Used  Used  Used  Not used  Used | 2160  1000  3.95  81  5600 | 100  96  1.92  1.4  68 | 160°  129°  9°  70°  90° | 6  3  2  6  6 | 320×288  320×320  352×256  352×286  100×76 | 433×433  400×400  400×296  400×325  380×289 | 36  25  14  24  233 |

*DW* diffusion-weighted, *EPI* echo planar imaging, *GRE* gradient recall echo, *HASTE* half fuorier single-shot turk spine-echo, *IP* in-phase, *MRI* magnetic resonance imaging, *OP* opposed-phase, *TE* echo time, *TR* repetition time, *TSE* turbo spin-echo, *VIBE* volume interpolated breath-hold examination, *2D* two-dimensional, *3D* three-dimensional

Supplementary Material 2 Definition of each MR imaging feature in this study

| MR imaging feature | Definitions |
| --- | --- |
| LI-RADS major imaging features |  |
| Nonrim arterial phase hyperenhancement | Nonrim-like enhancement in arterial phase unequivocally greater in whole or in part than liver |
| Nonperipheral “washout” | Nonperipheral visually assessed temporal reduction in enhancement in whole or in part relative to composite liver tissue from earlier to later phase resulting in hypoenhancement in the portal venous phase |
| Enhancing capsule | Smooth, uniform, sharp border around most or all of an observation, unequivocally thicker or more conspicuous than fibrotic tissue around background nodules, and visible as enhancing rim in PVP, DP, or TP |
| LI-RADS ancillary features (favoring HCC in particular) |  |
| Non-enhancing capsule | Capsule appearance not visible as an enhancing rim |
| Nodule-in-nodule architecture | Presence of smaller inner nodule within and having different imaging features than larger outer nodule |
| Mosaic architecture | Presence of randomly distributed internal nodules or compartments, usually with different imaging features |
| Fat in mass, more than adjacent liver | Excess fat within a mass, in whole or in part, relative to adjacent liver |
| Blood products in mass | Intralesional or perilesional hemorrhage in the absence of biopsy, trauma or intervention |
| LI-RADS ancillary features (favoring malignancy, not HCC in particular) |  |
| Transitional phase hypointensity | Intensity in the transitional phase unequivocally less, in whole or in part, than liver |
| Restricted diffusion | Intensity on DWI, not attributable solely to T2 shine-through, unequivocally higher than liver and/or ADC unequivocally lower than liver |
| Mild-moderate T2 hyperintensity | Intensity on T2WI mildly or moderately higher than liver and similar to or less than non-iron-overloaded spleen |
| Corona enhancement | Periobservational enhancement in late arterial phase or early PVP attributable to venous drainage from tumor |
| Fat sparing in solid mass | Relative paucity of fat in solid mass relative to steatotic liver OR in inner nodule relative to steatotic outer nodule |
| Hepatobiliary phase hypointensity | Intensity in the hepatobiliary phase unequivocally less, in whole or in part, than liver |
| Iron sparing in solid mass | Paucity of iron in solid mass relative to iron-overloaded liver OR in inner nodule relative to siderotic outer nodule |
| Tumor in vein | Unequivocal enhancing soft tissue in vein, regardless of visualization of parenchymal mass |
| Non-LIRADS imaging features |  |
| Nonsmooth tumor margin | An irregular margin that had budding portion at the tumor periphery protruding into the liver parenchyma ^[12]^ |
| Peritumoral hypointensity on HBP | An irregular, wedge-shaped or flamelike hypointense area of liver parenchyma surrounding the tumor on HBP ^[11]^ |
| Incomplete tumor capsule | A capsule only partly surrounding the tumor border ^[17]^ |
| Satellite nodule | The lesion smaller than 2 cm with similar MR imaging features and located in the uninvolved liver parenchyma within 2 cm of the main tumor ^[16]^. |

Unless otherwise indicated, all definitions of MR imaging features refer to the LI-RADS v2018 guideline ^[14]^

*ADC* apparent diffusion coefficient, *DP* delayed phase, *DWI* diffusion-weighted imaging, *HBP* hepatobiliary phase, *LI-RADS* Liver Imaging Reporting and Data System, *MR* magnetic resonance, *PVP* portal venous phase, *TP* transitional phase, *T2WI* T2-weighted imaging
